# Targeting MUC1-C inhibits the AKT-S6K1-elF4A pathway regulating TIGAR translation in colorectal cancer

**DOI:** 10.1186/s12943-017-0608-9

**Published:** 2017-02-02

**Authors:** Rehan Ahmad, Maroof Alam, Masanori Hasegawa, Yasumitsu Uchida, Omar Al-Obaid, Surender Kharbanda, Donald Kufe

**Affiliations:** 1 Colorectal Research Center, Department of Surgery, King Khalid University Hospital College of Medicine, King Saud University, PO BOX 7805 (37), Riyadh, Saudi Arabia; 2 Dana-Farber Cancer Institute, Harvard Medical School, 450 Brookline Ave, Boston, MA 02215 USA; 3 Genus Oncology, Boston, MA 02115 USA; 4 Present Address: Department of Urology, Keio University School of Medicine, Shinanomachi 35, Shinzyuyku-ku, Tokyo, 160-8582 Japan

**Keywords:** MUC1-C, AKT, S6K1, TIGAR, Colorectal cancer

## Abstract

**Background:**

Colorectal cancer is third most common malignancy and is the second most common cause of cancer-related death. The MUC1 heterodimeric protein is aberrantly overexpressed in colorectal cancer and has been linked to poor outcomes in this disease. Here, we investigate the effects of the MUC1-C subunit inhibitor (GO-203), which disrupts MUC1-C homo-oligomerization, on human colorectal cancer cells.

**Methods:**

TIGAR mRNA level was determined using qRT-PCR. Western blotting was used to measure TIGAR protein level and AKT-mTOR-S6K1 pathways. Reactive oxygen species and apoptosis were measured by flow cytometry. Effect of MUC1-C peptide, GO-203 was studied on colorectal xenograft tumors. Immunohistochemistry was utilized for TIGAR staining.

**Results:**

Treatment of MUC1-overexpressing SKCO-1 and Colo-205 colon cancer cells with GO-203 was associated with downregulation of the TP53-inducible glycolysis and apoptosis regulator (TIGAR) protein. TIGAR promotes the shunting of glycolytic intermediates into the pentose phosphate pathway and thus is of importance for maintaining redox balance. We show that GO-203-induced suppression of TIGAR is mediated by inhibition of AKT and the downstream mTOR pathway. The results also demonstrate that targeting MUC1-C blocks eIF4A cap-dependent translation of TIGAR. In concert with these results, GO-203-induced suppression of TIGAR was associated with decreases in GSH levels. GO-203 treatment also resulted in increases in reactive oxygen species (ROS) and loss of mitochondrial transmembrane potential. Consistent with these results, GO-203 inhibited the growth of colon cancer cells in vitro and as xenografts in nude mice. Inhibition of MUC1-C also downregulated TIGAR expression in xenograft tissues.

**Conclusions:**

These findings indicate that MUC1-C is a potential target for the treatment of colorectal cancer. Colorectal cancer patients who overexpress MUC1-C may be candidates for treatment with the MUC1-C inhibitor alone or in combination therapy with other agents.

## Background

Colorectal cancer (CRC) is the third most common cancer in the world. CRC is the fourth leading cause of cancer-related mortality, causing over 600,000 deaths per year. Colorectal cancer is the most common cancer in 40 European countries [1]. A large number of genetic and epigenetic alterations that lead to malignant transformation have been identified in colorectal cancer. These include alterations in genes involved in chromosomal and microsatellite instability. More than 50% of newly diagnosed colorectal cancer patients present with some type of metastasis [1]. The Wnt/β-catenin signaling pathway is of importance for the development of colorectal cancer with more than 80% mutation in APC (adenomatous polyposis coli) and about 5% activating mutations in β-catenin [2, 3]. The proliferation and differentiation of intestinal stem and progenitor cells is regulated by Wnt signaling. Mutations in APC and β-catenin are among the earlier events that lead to hyperplasia in the intestinal crypts leading to colorectal cancer development [4, 5]. Moreover, Wnt signaling is required for colorectal cancer progression and metastasis [6].

MUC1 (mucin 1) is a heterodimeric glycoprotein expressed on the apical surface of normal epithelium and is overexpressed in diverse cancers [7]. MUC1 is translated as a single polypeptide and is processed by auto-cleavage into two subunits that form a stable heterodimeric complex at the cell membrane. The N-terminal subunit of MUC1 (MUC1-N) contains variable numbers of 20 amino acid tandem repeats that are glycosylated on serine and threonine. The MUC1 C-terminal subunit (MUC1-C) is composed of a 58 amino acid extracellular domain, a 28 amino acid transmembrane domain and a 72 amino acid cytoplasmic domain (MUC1-CD) [7]. MUC1-N is attached to MUC1-C on the apical border of normal epithelial cells. Shedding of MUC1-N from the cell surface frees MUC1-C to function as a putative receptor inside the cell in response to stress [7]. Importantly, the MUC1-C cytoplasmic domain is sufficient for transformation [8]. MUC1-C interacts with diverse effectors, such as β-catenin [9], IKK β [10], and NFkB p65 [11], which have been linked to transformation. Wnt and NFkB signaling pathways are known to be activated in colorectal cancer [12, 13]. Incorporation of MUC1-C into the outer mitochondrial membrane also inhibits stress-induced loss of the mitochondrial transmembrane potential [14]. Overexpression of MUC1 in human cancers blocks the induction of apoptosis and necrosis in response to DNA damaging agents [15], reactive oxygen species [16, 17] and hypoxia [18]. MUC1 is known to be overexpressed in human colorectal cancer cells [19]. The MUC1 oncoprotein is predictive for tumor progression in colorectal cancers [20], including those related to HNPCC (Hereditary nonpolyposis colorectal cancer) [21]. The overexpression of MUC1 predominates at the invasive front [20]. A positive correlation has been described between mucin secretion, proliferation, invasiveness, metastasis and poor prognosis [21, 22]. Overexpression of MUC1 is more pronounced at the deepest tumor invasive portion, lymphatic and venous invasion, and lymph node and liver metastasis [23]. Correlation of MUC1 with poor prognosis has been reported in mismatch repair gene colorectal tumors but not in MLH1 negative tumors or in Lynch syndrome (HNPCC) [24]. Research on MUC1 as a target in colorectal cancer has been focused on the development of a vaccine [25, 26]. However, there are no available small molecules or peptides that target MUC1 for treating MUC1-positive colorectal cancers. MUC1-C contains CQC residues in the cytoplasmic domain that are important for its homodimerization and function as an oncoprotein [27]. Accordingly, direct inhibitors of MUC1-C, designated GO-201/GO-203 have been developed to block the CQC motif [28].

Protein translation is a tightly regulated process that is regulated by translation initiation. This initiation step is controlled by the initiation factors (elF4F) complex at the ribosomal recruitment [29]. The elF4F complex is formed by the binding of elF4E to the 5′ cap of mRNAs and recruitment of elF4G and elF4A. The PI3K-AKT signaling pathway is a major regulator of protein translation which is upstream to the mammalian target of rapamycin complex 1 (mTORC1). mTORC1 activates 40S ribosomal protein S6 kinases (S6Ks) which contribute to cap-dependent translation that, in turn, enhance the eIF4A RNA helicase activity [29]. S6K induces degradation of the tumor suppressor programmed cell death protein 4 (PDCD4), which is an eIF4A inhibitor [30]. eIF4A initiates translation by unwinding highly structured 5′ untranslated regions (UTRs) in mRNAs, such as those encoding cyclin D1 and MYC [31]. In this way, tumor cells can regulate translation in response to growth signals through mTORC1-induced activation of the eIF4A RNA helicase function.

The present study demonstrates that treatment of human colorectal cancer cell lines with the MUC1-C inhibitor GO-203 is associated with downregulation of the AKT→mTOR pathway and suppression of cap-dependent translation of the TIGAR (TP53-induced glycolysis and apoptosis regulator) protein. Targeting MUC1-C with GO-203, in turn, results in disruption of redox balance and inhibition of growth, supporting the notion that MUC1-C is a potential target for the treatment of colorectal cancer.

## Methods

### Cell culture

Human SW480 and LOVO colorectal cancer cells were grown in DMEM (Cellgro) containing 10% heat-inactivated fetal bovine serum, 100 μg/ml streptomycin, 100 units/ml penicillin and 2 mmol/l L-glutamine. Human COLO-205 colon cancer cells were cultured in RPMI (ATCC) containing 10% heat-inactivated fetal bovine serum 100 μg/ml streptomycin, 100 units/ml penicillin and 2 mmol/L-glutamine. Authenticity of the cells was confirmed by short tandem repeat (STR) analysis. Mycoplasma contamination testing was done using kit (MycoAlert, Lonza). The cells were treated with GO-203 and control peptide CP-2 (Genus Oncology, Boston) as described [7]. Cells were treated with the PI3K inhibitor LY294002 (Cayman Chemical Company), rapamycin (Cell Signaling Technology), AKT inhibitor GSK690693 (Selleck Chemicals) and Silvestrol was gift from Prof John A. Porco, Jr [32].

### Quantitative RT-PCR

For qRT-PCR analysis, cDNA synthesis was performed with 1 μg total RNA using the High Capacity Reverse Transcription Kit (Applied Biosystems). The cDNA samples were amplified using the SYBR Green qPCR assay kit (Applied Biosystems) and the StepOnePlus real time PCR system (Applied Biosystems). TIGAR Primers used for qRT-PCR: Forward 5′ CTCCAGTGATCTCATGAG 3′ Reverse 5′-AGACACTGGCTGCTAATC 3′. Statistical significance was determined by the Student’s *t* test.

### Western blotting

Whole cell lysates were prepared as described [11]. Soluble proteins were analyzed by immunoblotting with anti-MUC1-C (Ab5; Thermo Scientific), anti-TIGAR (Abcam), anti-p-Akt, anti-Akt, anti-p-S6K1, anti-S6K1, anti-PDCD4 (Cell signaling Technology) and anti-β-actin (Sigma). Reactivity was detected with horseradish peroxidase-conjugated secondary antibodies and chemiluminescence (GE healthcare).

### Cell viability assay

To assess cell viability, 20,000–30,000 cells were plated and cultured for 48 h. GO-203 and CP-2 were added at 5 μM every day for six days. Cell viability was measured by trypan blue exclusion.

### Reactive oxygen species (ROS) measurement

Cells were incubated with 5 μM DCFH-DA (Molecular Probes) for 20 min at 37 °C. Fluorescence of oxidized DCF was measure at an excitation of 480 nm and an emission at 525 nm.

### Determination of Glutathione (GSH) levels

The levels of GSH in the cells were determined according to the method [33] based on the formation of 2-nitro-5-tiobenzoic acid from DTNB in the presence of GSH. In brief, 25 μl of trichloroacetic acid (15%) was added to 50 μl of the homogenate, followed by centrifugation at 13,000 × *g* for 5 min at 4 °C. A supernatant aliquot (50 μl) was mixed with 50 μl of 3.4 mM ethylenediaminetetraacetic acid (EDTA) dissolved in PBS, 1 ml of PBS, and 250 μl of DTNB in PBS (20 mg/ml). The absorbance was measured at 412 nm after 15 min and compared to a standard curve of GSH (0.01–0.5 mM).

### Mitochondrial membrane potential analysis

Mitochondrial membrane potential was measured using the JC1 Mitochondrial Membrane Potential Assay Kit (Abcam) following the manufacturer’s instructions.

### Analysis of apoptosis

Cells were fixed with 80% ethanol and incubated in PBS containing 40 μg/ml RNase and 40 μg/ml propidium iodide. Sub-G1 analysis was determined by flow cytometry.

### Colon tumor xenografts models

Four- to 6-week-old BALB/c *nu/nu* male/female mice were injected subcutaneously with 1 × 10^7^ Colo-205 or SKCO-1 cells in the flank. When tumors were detectable, the mice were pair-matched into control and treatment groups of 8 mice each. Mice were excluded if the tumors were not within 15% of the mean volume. PBS (control vehicle), 18 mg/kg GO-203, or 18 mg/kg CP-2 (peptides dissolved in PBS) were administered daily by intraperitoneal injection for 28 days. Tumor volume (*V*) was calculated using the formula *V* = *L* ^2^ × *W*/2, where *L* and *W* are the larger and smaller diameters, respectively. For SKCO-1 tumor xenografts, Groups of 8 mice each were treated with PBS (Control), 6 mg/kg GO-203 each day for 5 days intravenously (iv), or 3 mg/kg GO-203 each day for 5 days iv each week for two weeks.

### Immunohistochemistry

Paraffin-embedded blocks of xenograft tumor tissues were deparaffinized in xylene and rehydrated using a graded ethanol series. Antigen was retrieved by boiling the slides in a microwave oven for 15 min in 0.01 mol/l citrate buffer (pH 6.0). Endogenous peroxidase was blocked with a 3% H_2_O_2_-methanol solution, and the slides were incubated in 10% normal goat serum for 30 min to prevent non-specific staining. The tissue sections were then incubated overnight at 4 °C with primary antibody [TIGAR sc-166291 (1:100 dilution); Santa Cruz Biotechnology, Dallas, TX, USA]. The standard biotin-streptavidin-peroxidase method was used and the sections were lightly counterstained with hematoxylin. The expression of TIGAR in tumor tissues was analyzed using the eSlide capture device (ScanScope CS, Aperio Technologies Inc., Vista, CA, USA).

## Results

### Targeting MUC1-C inhibits AKT-mTORC-S6K1 signaling

**Fig. 1:**
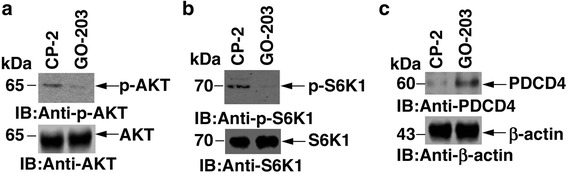
GO-203 inhibits AKT-mTORC-S6K1 translation pathway. **a**-**c** SKCO-1 cells were treated each day with 5 μM GO-203 or CP-2 for three days and lysates were immunoblotted with indicated antibodies

GO-203 inhibits MUC1-C homodimerization which blocks its oncogenic function [28]. The effect of GO-203 has not been studied on the AKT-mTORC-S6K1 translation pathway. Treatment of SKCO-1 cells with GO-203 was associated with downregulation of p-AKT (Fig. 1a). The PI3K-AKT pathway is a major protein synthesis regulator [29]. This pathway is also upstream of the mTORC1 (mammalian target of rapamycin complex 1). mTORC1 transmit positive signal to activate p70 S6 kinase and participate in the inactivation of the elF4E inhibitor, 4E-BP1 [29]. GO-203 inhibited the activation of S6K1 in SKCO-1 cells (Fig. 1b). Activation of S6K1 leads to the phosphorylation of PDCD4 at Ser 67 and is subsequently degraded by the ubiquitin ligase SCF (betaTRCP) [30]. Regulated degradation of PDCD4 in response to oncogene activation allows efficient protein synthesis and cell growth. Activation of the elF4A RNA helicase is crucial for the initiation of translation. To understand the potential role of elF4A, we studied the effect of GO-203 on the expression of the tumor suppressor programmed cell death protein 4 (PDCD4), which is known to block elF4A RNA helicase activity [30]. GO-203 treatment of SKCO1 cells inhibited the degradation of PDCD4 (Fig. 1c). These findings demonstrate that targeting MUC1-C with GO-203 inhibits AKT-mTORC-S6K1 signaling in colorectal cancer cells.

### Inhibition of MUC1-C blocks TIGAR translation

**Fig. 2:**
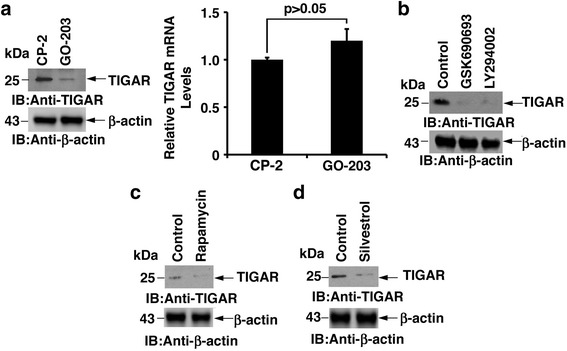
GO-203 inhibits TIGAR translation. **a** SKCO-1 cells were treated each day with 5 μM GO-203 or CP-2 for three days and lysates were immunoblotted with indicated antibodies (*Left*). TIGAR mRNA levels were determined by qRT-PCR. The results are expressed as relative TIGAR mRNA levels (mean ± sd of three determinations) compared with that obtained for GAPDH as a control (*Right*). **b** SKCO-1 cells were treated with the PI3K inhibitor LY294002 (50 μM) and Akt inhibitor GSK690693 (10 μM) for 48 h. Cell lysates were immunoblotted with indicated antibodies. **c** SKCO-1 cells were treated with Rapamycin (100 nM) for 24 h. Cell lysates were immunoblotted with indicated antibodies. **d** SKCO-1 cells were treated with Silvestrol (100 nM) for 24 h. Lysates were immunoblotted with indicated antibodies

TIGAR (TP53-induced glycolysis and apoptosis regulator) has been reported to be highly expressed in human colon cancers with tumorigenic phenotype [34, 35]. Targeting MUC1-C in hematologic malignancies has been reported to inhibit TIGAR at the protein level [36, 37]. The mechanism for TIGAR downregulation is still unknown. To further understand TIGAR protein inhibition, SKCO-1 cells were treated with GO-203 and control peptide CP-2. GO-203 treatment was associated with a marked depletion of TIGAR levels (Fig. 2a, Left). Interestingly, there was no significant change in TIGAR mRNA levels between GO-203 and CP-2 treated cells (Fig. 2a, Right), supporting a potential translational mechanism. To determine whether the AKT-mTORC-S6K pathway is involved in TIGAR protein translation, SKCO1 cells were treated with PI3K inhibitor, LY294002 and Akt inhibitor, GSK690693. Both inhibitors significantly reduced TIGAR protein levels (Fig. 2b), indicating that PI3K-AKT is involved in TIGAR protein synthesis. To assess the potential role of mTORC, Rapamycin, an inhibitor of mTORC was used to measure TIGAR levels. Indeed, Rapamycin treatment inhibited TIGAR levels (Fig. 2c). Silvestrol is an inhibitor of the translation initiation factor elF4A RNA helicase [32]. Silvestrol-treated SKCO1 cells resulted in a significant downregulation of TIGAR protein (Fig. 2d). These results indicate that GO-203 downregulates TIGAR protein synthesis by inhibiting the PI3K-AKT-S6K1 pathway.

### Effect of GO-203 on glutathione and reactive oxygen species (ROS)

**Fig. 3:**
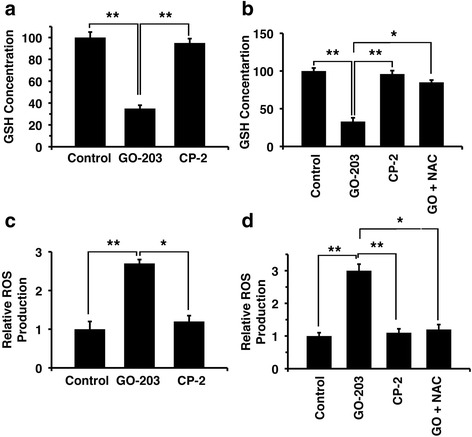
GO-203 disrupts the redox balance. **a** SKCO-1 cells were treated each day with 5 μM GO-203 or CP-2 for three days. These cells were harvested and analyzed for GSH levels using the DTNB method. **b** SKCO-1 cells were treated each day with 5 μM GO-203 or CP-2 for three days. The GO-203 treated cells were also incubated with 5 mM NAC. The cells were analyzed for GSH levels. **c** SKCO-1 cells were treated with 5 μM GO-203 or CP-2 for three days. The cells were incubated with DC-FDA, and the fluorescence of oxidized DCF was measured by a microplate reader. **d** The GO-203 treated cells were also incubated with 5 mM NAC and ROS generation was measured using DC-FDA. * *p* < 0.05, ** *p* < 0.01

Treatment of SKCO-1 cells with GO-203 significantly decreased GSH levels. In contrast, the inactive control peptide CP-2 had no apparent effect on GSH levels (Fig. 3a). N-acetylcysteine (NAC) is a precursor for GSH biosynthesis. We therefore investigated whether NAC alters the GO-203-induced disruption of redox balance. Indeed, the addition of NAC with GO-203 blocked GSH depletion (Fig. 3b). As reported previously [16], MUC1-C blocks the disruption of redox balance and MUC1-C silencing is associated with increases in reactive oxygen species (ROS) production. GO-203 treatment of SKCO-1 cells was associated with a more than 2-fold increase in ROS production; however, CP-2 had little effect (Fig. 3c). In addition, GO-203-induced ROS production was blocked by NAC (Fig. 3d). These results indicate that MUC1-C inhibition leads to depletion of GSH and induction of ROS production. Moreover, both of these effects of GO-203 are reversed by NAC.

### Effect of GO-203 on mitochondrial membrane potential and apoptosis

**Fig. 4:**
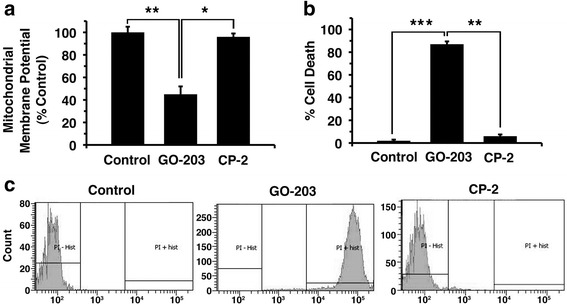
**a** SKCO-1 cells were treated with GO-203 and CP-2 for three days then mitochondrial membrane potential (JC-1) was measured. The results are expressed as the percentage (mean ± SD of 3 determinations) compared with that obtained for the control. **b**-**c** SKCO-1 cells were incubated with propidium iodide and analyzed by flow cytometry. The results are expressed as the percentage of cells with apoptosis. * *p* < 0.01, ** *p* < 0.01, *** *p* < 0.001

Disruption of redox balance leads to the loss of mitochondrial membrane potential and apoptosis [38]. In this regard, treatment of SKCO-1 cells with GO-203 resulted in a significant decrease in mitochondrial membrane potential (Fig. 4a). To further assess the effects on cell death, SKCO-1 cells were treated with GO-203 for 3 days. GO-203 treatment was associated with uptake of propidium iodide due to loss of cell membrane integrity (Fig. 4b-c). GO-203 induced approximately 80% death of SKCO-1 cells (Fig. 4b). By contrast, CP-2, had little effect on cell viability.

### Effect of GO-203 on colorectal cancer cell growth

**Fig. 5:**
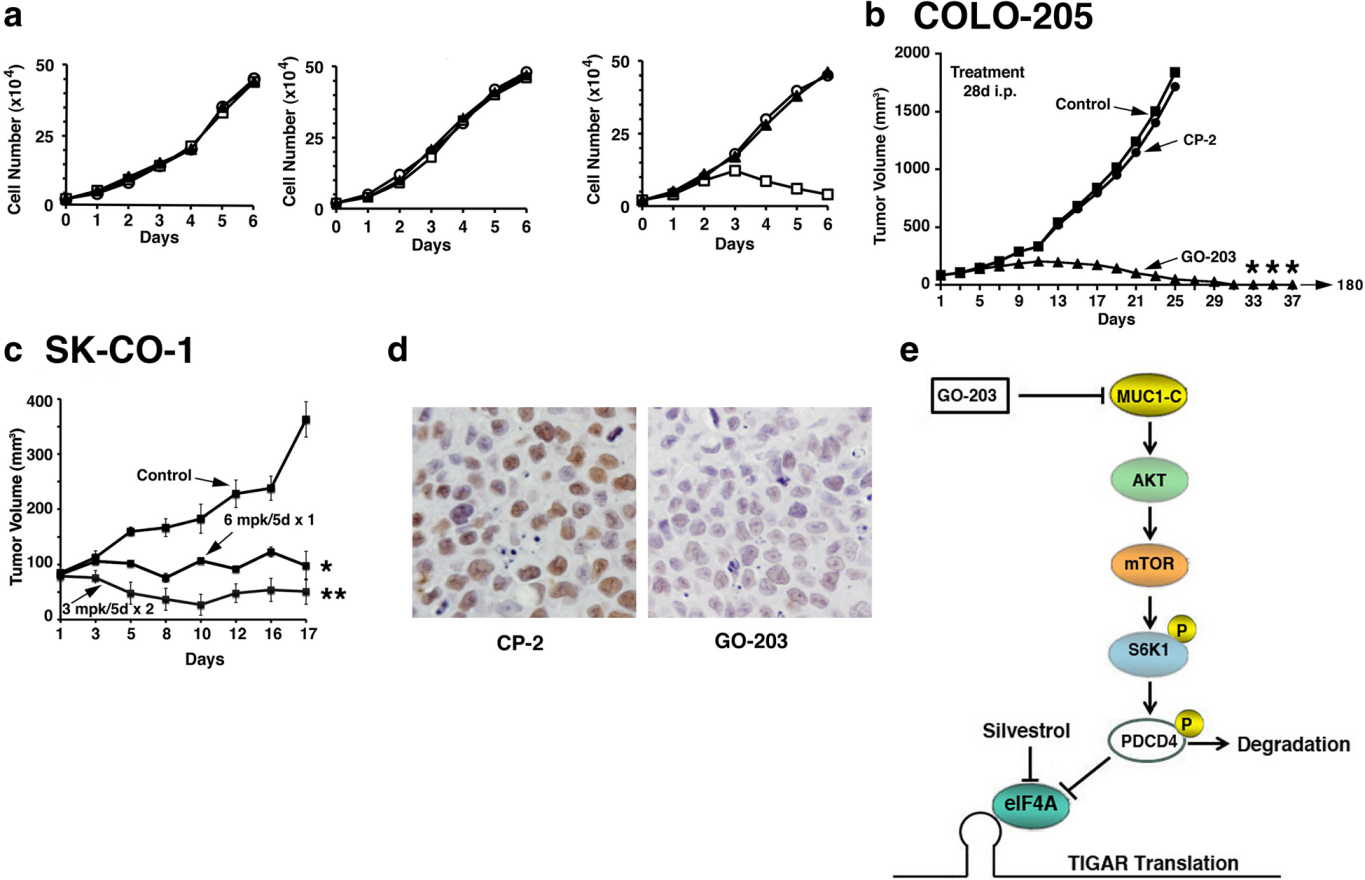
GO-203 inhibits cell proliferation and regress tumor growth. **a** SW480 (*Left*), LoVo (*Center*) and COLO-205 (*Right*) cells were left untreated, or were treated with 5 μM GO-203 and CP-2 each day for the indicated days. Viable cell numbers were determined by trypan blue exclusion and is expressed as the mean ± sd of three determinations. Control: Circle; GO-203: Square; CP-2: Triangle. **b**-**c** BALB/c nu/nu mice were injected subcutaneously in the flank with 1 × 107 COLO-205 (**b**) or SK-CO-1 (**c**) cells. The mice were pair matched when the tumors were ~100 mm3. **b** Treatment groups consisted of 8 mice injected intraperitoneally (ip) with PBS (vehicle control) (*Square*), 18 mg/kg GO-203(*Circle*) or 18 mg/kg CP-3 (*Triangle*) each day for 28 days. **c** Treatment groups consisted of 8 mice injected iv with PBS, 6 mg/kg GO-203 each day for 5 days, or 3 mg/kg GO-203 each day for 5 days each week for 2 weeks. Tumor measurements were performed as indicated and mice were weighed twice weekly. There was no weight loss in any of the groups. **d** Harvested tissues were stained for TIGAR expression by immunohistochemistry. **e** Schema depicting the proposed mechanism. * *p* < 0.05, ** *p* < 0.01, *** *p* < 0.001 as compared to control

Treatment of MUC1-negative SW480 and LOVO cells with GO-203 had no effect on cell growth (Fig. 5a). By contrast, GO-203 significantly inhibited MUC1-positive COLO-205 cell growth and decreased viable cell number (Fig. 5a). GO-203 has also been reported to inhibit MUC1- positive SKCO-1 cell proliferation [39]. These findings demonstrate that the effects of GO-203 on cell growth and survival are selective for MUC1-expressing cells. Moreover, GO-203 inhibits MUC1 positive colorectal cancer cell proliferation by decreasing intracellular GSH levels and enhanced ROS production.

### GO-203 induces regression of colon tumors in xenografts models

To extend these results obtained from in vitro experiments, we established subcutaneous COLO-205 colon tumor xenografts (~90 mm^3^) in the flanks of nude mice. Groups of 8 mice each were treated intraperitoneally (ip) with PBS (Control), 18 mg/kg GO-203 or 18 mg/kg CP-2 each day for 28 days (Fig. 5b). Compared to the control group, growth of the COLO-205 tumors was significantly inhibited in the GO-203-treated mice. Moreover, these tumors regressed completely by the end of treatment (day 28) and there was no evidence for regrowth by day 180 (Fig. 5b). In contrast to the anti-tumor activity of GO-203, treatment with CP-2 had no effect on COLO-205 tumor growth (Fig. 5b). Importantly, GO-203 was not associated with loss of body weight or other apparent toxicities. Studies were also performed on established subcutaneous SK-CO-1 tumor xenografts (~90 mm^3^) to explore other GO-203 dose-schedules (Fig. 5c). Groups of 8 mice each were treated with PBS (Control), 6 mg/kg GO-203 each day for 5 days intravenously (iv) or 3 mg/kg GO-203 each day for 5 days iv each week for two weeks. The results demonstrate that, as compared to the control group, growth of tumors in the GO-203-treated mice was inhibited significantly with both dose-schedules (Fig. 5c). These findings indicate that GO-203 is effective in inhibiting growth and survival of MUC1-positive colorectal cancer cells in mouse xenograft models. Tumor tissues from the GO-203 and CP-2 treated mice were harvested and stained for TIGAR. TIGAR levels were decreased substantially by GO-203 treatment, further confirming that inhibition of MUC1-C function downregulates TIGAR expression (Fig. 5d).

## Discussion

MUC1 is expressed in colorectal tumors, predominantly in those with more aggressive disease [19–23]. However, there has been no direct proof that MUC1 is essential for colorectal cancer cell growth and survival. Protein synthesis is known to be regulated by PI3K-AKT signaling. AKT controls translation by activation of mTORC1 that results in the phosphorylation of p70S6 Kinase [40]. In turn, S6K phosphorylates and thereby induces the degradation of PDCD4, an inhibitor of eIF4A RNA helicase activity that regulates translation of proteins [30]. The present work demonstrates that targeting MUC1-C with GO-203 inhibits p-AKT in colorectal cancer cells. GO-203 further blocks the phosphorylation of S6K1. PDCD4 is a downstream target of S6K1, which undergoes phosphorylation, ubiquitination and degradation, contributing to efficient protein synthesis for growth and survival. GO-203 inhibited the degradation of PDCD4 which inhibits elF4A helicase activity. Hence these findings demonstrate that GO-203 regulates AKT-S6K1-elF4A signaling in colorectal cancer cells.

TIGAR protein has been reported to be overexpressed in colon cancer [34, 35]. Inhibition of MUC1-C in hematological malignancies downregulates TIGAR at the protein level [36, 37]. GO-203 treatment of colorectal cancer cells also resulted in the depletion of TIGAR protein without any change in mRNA levels. Inhibitors of PI3K and Akt downregulated TIGAR protein, providing evidence that these pathways are involved in the depletion of TIGAR. GO-203 is known to inhibit AKT phosphorylation [41]. GO-203 further blocks the phosphorylation of S6K1 in colorectal cancer cells. Rapamycin, an inhibitor for mTORC1 downregulated TIGAR protein levels. Similarly Silvestrol, an inhibitor for elF4A RNA helicase activity, also inhibits TIGAR protein levels. These studies thus demonstrate that GO-203 induced TIGAR downregulation is regulated by PI3K-AKT-S6K1 translation pathway.

Oxidized glutathione is converted to reduced glutathione by NADPH. With MUC1-C inhibition, NADPH would be depleted, with subsequent decreases in GSH levels and increases in ROS. Indeed, GO-203-induced alteration of redox balance was followed with significant loss of GSH levels. NAC treatment with GO-203 restored GSH levels and blocked increases in ROS. The addition of NAC was sufficient to replenish GSH levels. This finding is consistent with other reports showing that GO-203 induces ROS production [36, 37]. The electron transport chain present in the mitochondrial inner membrane generates ROS. MUC1-C translocates to the mitochondrial outer membrane in response to stress, where it attenuates a loss of mitochondrial membrane potential [14, 15]. Here, we demonstrate that the inhibition of MUC1-C results in a loss of the mitochondrial membrane potential. Targeting MUC1-C with GO-203 induced disruption of redox balance was associated with a substantial loss of mitochondrial membrane potential. In concert with this response, GO-203 was associated with loss of cell membrane integrity and induction of apoptosis.

MUC1-C is expressed in certain colorectal cancer cells [39]. The MUC1-C transforming function is dependent on homodimer formation, which is mediated by a CQC motif present in the cytoplasmic domain of MUC1-C [27]. GO-203 is an inhibitor of MUC1-C, blocks MUC1-C oligomerization and inhibits the function of MUC1-C as an intracellular signaling protein [28]. This MUC1 inhibitor has been reported to be effective in inhibiting cell proliferation in-vitro and in xenografts models of breast [28], prostate [42], lung [43] and certain hematologic malignancy [44]. Here, we show that treatment of MUC1-C-positive COLO-205 cells with GO-203 is associated with growth inhibition. By contrast, the MUC1-C inhibitor GO-203 had no apparent effect on proliferation of MUC1-C negative colorectal cancer cell lines. The specificity of GO-203 on MUC1-C-positive colorectal cancer cells is significant, because GO-203 binds to the CQC motif of MUC1-C and directly blocks MUC1-C function. The control peptide CP-2, in which the CQC motif has been changed to an AQA motif [28], does not bind to MUC1-C and therefore has no effect on the growth of MUC1-positive colorectal cancer cells.

Treatment of COLO-205 tumors in nude mice responded to GO-203 treatment with an initial slowing of growth. At 28 days of GO-203 treatment COLO-205 tumor growth was also slowed after discontinuing dosing and the tumors regressed completely. Similarly, in the SKCO-1 tumor xenograft model, regression of tumor was found to be evident in response to a different dose schedule. These findings indicate that GO-203 is effective in inhibiting growth and survival of MUC1-positive colorectal cancer cells in xenograft models. The findings also support a GO-203 dose-response effect with regression of tumors at higher doses (18 mg/kg) administered for longer periods (28 days). The effectiveness of GO-203 in colorectal tumor xenografts can be linked to MUC1 and TIGAR expression in human colon cancers. Indeed, TIGAR has been reported to be highly expressed in human colon cancers with a tumorigenic phenotype [34, 35]. Confirming this correlation between MUC1 and TIGAR, inhibition of MUC1-C function downregulates TIGAR expression in xenograft tissues.

This study supports the importance of MUC1-C in supporting the growth and survival of MUC1-C positive colorectal cancer cells. There has been no previous evidence that MUC1-C represents a potential target for the treatment of colorectal cancer. Importantly, MUC1-C is known to interact with β-catenin [9] during cell adhesion. MUC1-C is also known to bind IKKβ [10] and Rel A p65 [11] and contributes to constitutive NFκB activation in cancer cells. It has been reported earlier that NFκB is constitutively activated in colorectal carcinoma which play important role in angiogenesis and promoting tumor growth [13]. Therefore, it is possible that MUC1-C may be involved in activating β-catenin and NFκB signaling pathways and thereby colorectal cancer progression and hence targeting MUC1-C with GO-203 could also inhibit these pathways in colorectal cancer cells.

## Conclusions

Based on these results, we conclude that the targeting MUC1-C function by GO-203 downregulates the AKT-mTORC-S6K1 translation pathway that leads to the depletion of TIGAR translation. GO-203 further downregulates GSH levels, increases ROS production, decreases mitochondrial membrane potential and disrupts cell membrane integrity leading to cell death. Hence, inhibition of MUC1-C blocks the cellular proliferation and survival of colorectal cancer cells leading to tumor regression in colorectal xenografts models and inhibits TIGAR expression in xenograft tissues. Therefore, colorectal cancer patients who overexpress MUC1-C may be candidates for treatment with the MUC1-C inhibitor alone or in combination therapy with other agents.

## Abbreviations

CRC: Colorectal cancer
DCFDA: 2′,7′-dichlorodihydrofluorescein diacetate
DMSO: Dimethyl sulfoxide
DTNB: 5,5-dithio-bis-(2-nitrobenzoic acid)
EDTA: ethylenediaminetetraacetic acid
GSH: Glutathione
MTT: 3-(4, 5-dimethylthiazolyl-2)-2, 5diphenyltetrazolium bromide
NAC: N-acetylcysteine
PI: Propidium iodide
ROS: Reactive oxygen species
